# Hypoxia Inducible Factor (HIF) transcription factor family expansion, diversification, divergence and selection in eukaryotes

**DOI:** 10.1371/journal.pone.0179545

**Published:** 2017-06-14

**Authors:** Allie M. Graham, Jason S. Presnell

**Affiliations:** Department of Biology, University of Miami, Coral Gables, Florida, United States of America; Laboratoire Oceanologique de Banyuls sur Mer, FRANCE

## Abstract

Hypoxia inducible factor (HIF) transcription factors are crucial for regulating a variety of cellular activities in response to oxygen stress (hypoxia). In this study, we determine the evolutionary history of HIF genes and their associated transactivation domains, as well as perform selection and functional divergence analyses across their four characteristic domains. Here we show that the HIF genes are restricted to metazoans: At least one HIF-α homolog is found within the genomes of non-bilaterians and bilaterian invertebrates, while most vertebrate genomes contain between two and six HIF-α genes. We also find widespread purifying selection across all four characteristic domain types, bHLH, PAS, NTAD, CTAD, in HIF-α genes, and evidence for Type I functional divergence between HIF-1α, HIF-2α /EPAS, and invertebrate HIF genes. Overall, we describe the evolutionary histories of the HIF transcription factor gene family and its associated transactivation domains in eukaryotes. We show that the NTAD and CTAD domains appear *de novo*, without any appearance outside of the HIF-α subunits. Although they both appear in invertebrates as well as vertebrate HIF- α sequences, there seems to have been a substantial loss across invertebrates or were convergently acquired in these few lineages. We reaffirm that HIF-1α is phylogenetically conserved among most metazoans, whereas HIF-2α appeared later. Overall, our findings can be attributed to the substantial integration of this transcription factor family into the critical tasks associated with maintenance of oxygen homeostasis and vascularization, particularly in the vertebrate lineage.

## Introduction

The maintenance of oxygen homeostasis is a critical biological constraint that requires coordinated regulation of a variety of genes, especially for metazoans whom rely mostly on aerobic energy production [1,2]. In hypoxic conditions, situations where there is inadequate oxygen supply or low oxygen, genes involved in mitochondrial function, energy metabolism, oxygen binding and delivery, and hematopoiesis are activated [3]. During periods of reduced oxygen supply, the most profound changes in gene expression are mediated by transcription factors known as Hypoxia inducible factors (HIF) [4]. The HIF transcription factor family plays a crucial role in cellular response to low oxygen tension in a variety of organisms, and is frequently associated with adaptations to high altitude [5–12] and other oxygen limited environments [13]. The HIF-1 heterodimer is considered a “master-regulator” of oxygen homeostasis [14–16]. Members of the HIF family are also known for their roles in vasodilation, cell migration, signaling, and cell fate specification [17].

Members of the HIF gene family encode both alpha and beta subunits which generally form functional heterodimers to regulate transcription [15]. In humans there are three paralogs of the HIF-α subunit (HIF-1α, HIF-2α/EPAS, HIF-3α) and two paralogs of the HIF-β subunit (ARNT, ARNT2). ARNTL is closely related to ARNT, but mostly functions as the β subunit that dimerizes with CLOCK. Either HIF-1α or HIF-2α can heterodimerize with any of the HIF-β subunits to form functional HIF transcription factor complexes [18]. Across multiple species, hypoxic declining partial pressure of oxygen post-translationally activates the regulatory α-subunit of HIF, while normoxic conditions quickly lead to its degradation; thus, HIF activity is thought to be controlled at the level of its α-subunits [19].

HIF-α and HIF-β (ARNT) genes are a subfamily of the expansive bHLH+PAS containing gene family, and their proteins are characterized by the presence of an N-terminal bHLH DNA binding domain just upstream of two PAS domains [16]. In addition, α-subunits usually include an inhibitory domain called the oxygen-dependent degradation domain (ODDD), and an N-terminal transactivation domain (NTAD). A subset of HIF-α proteins, namely HIF-1α and HIF-2α (EPAS) are characterized by the presence of a C-terminal transactivation domain (CTAD) located at the C-terminal end of the protein [18]. These domains are considered critical to the overall function of HIF proteins: the bHLH domain contacts the core nucleotides of HIF-responsive elements [20], while bHLH and PAS domains together mediate both dimerization and sequence specific DNA binding [21,22]. The NTAD is thought to confer target specificity [23], while the CTAD is required for full HIF activity [24] and interactions with co-activators [25,26].

Although HIF genes have been recognized as key drivers for high altitude adaptation in human populations and other animals [27–33], studies investigating the broad evolutionary history of this gene family tend to have focused on lineage-specific evolution and lack a broad selection of non-bilaterian and bilaterian invertebrate species [9–12,34]. Here, we assessed the broad evolutionary history of the HIF gene family, with an emphasis on sampling taxa that have been excluded in previous studies, namely representatives from all four non-bilaterian phyla and species representing major groups of protostome lineages. To evaluate the expansion and diversification of the HIF gene family within eukaryotes, we used a combination of domain architecture characterization and phylogenetic analyses to identify and compare HIF genes across a wide sampling of genomes. We also investigated the separate evolutionary histories of the characteristic functional domains that characterize the HIF family. Furthermore, for the HIF-α group, we tested the functional domains for evidence of selection pressures and functional divergence to understand why the patterns of HIF gene family evolution were observed.

## Materials and methods

### HIF identification pipeline

To determine the genomic complement of HIF genes in a diverse range of eukaryotes, we searched for the presence of a combination of characteristic domains unique to HIF proteins in publicly available genomes of 44 eukaryotic species (S1 Table) including 31 metazoans, 11 unicellular amorpheans, and 2 bikonts. Inferred phylogenetic relationships for insects are from [35]; metazoans [36,37]; and unicellular amorpheans and bikonts [38]. Hidden Markov Models for the different domains were downloaded from the Pfam database: basic helix-loop-helix (bHLH; PF00010), PAS (PF00989), HIF-NTAD (HIF-1; PF11413) and HIF-1 alpha C terminal transactivation domain (HIF-CTAD; PF08778). We used the *hmmsearch* command from the HMMER 3.0 program [39], along with perl scripts to identify proteins that contained the following combination of domains: (1) bHLH domain, (2) bHLH+PAS, (3) bHLH+PAS + NTAD and (4) bHLH+PAS + NTAD +CTAD. We additionally searched the genomes using each domain separately. In some instances, we found genes that contained specific domains (e.g., CTAD) but were not previously identified in the pipeline due to high sequence divergence of the bHLH domain. These sequences were added to the collection of sequences identified through the search pipeline. Additionally, we obtained putative HIF-α sequences for the non-bilaterians *Nematostella vectensis* and *Trichoplax adhaerens* from previous reports [34,40]. This curated output was used for subsequent analyses. A list of the sequence IDs along with the genomic database from where we obtained the sequences is listed in S1 Table.

### Phylogenetic analyses

A perl script was used to extract the relevant domains from the HMMER datasets with their protein location information. A multiple sequence alignment was built with MUSCLE [41] using the default parameters. The alignment contained the concatenated bHLH and PAS domains identified in the eukaryotic genomes. JTT+G was determined to be the best fit substitution model for the alignment using ProtTest 1.4 [42]. Maximum Likelihood analysis was performed using PhyML [43] with bootstraps (100 replicates). Bayesian analysis were performed using BEAST v 1.7.5 [44] at 10,000,000 chain-length, and 1,000 burn-in. Trees were visualized and edited in FigTree 1.4.0 [45] and on the EvolView server [46]. Script and datasets/alignments used are publicly available via github (https://github.com/amgraham07/HIF_eukaryote).

### Protein domain location and selection analyses–site specific and alignment wide

Selection analyses were performed on the four concatenated domains (bHLH, PAS, NTAD and CTAD) both in the form of a protein alignment and codon alignment of the HIF-α members. This was not performed in the ARNTs due to their additional interaction with other bHLH-PAS gene families who have different functional responsibilities, thus their evolutionary history likely also represents selection pressures beyond those principally involved in oxygen-sensing. To identify past selection on individual codons, we used Single-Likelihood Ancestor Counting [SLAC], Fixed-Effects Likelihood [FEL], Mixed Effects Model of Evolution [MEME] and Fast-Unconstrained Baysian AppRoximation method [FUBAR] with default settings implemented in the Datamonkey web interface for the HYPHY package [47,48]. To avoid a high false-positive rate, due to the reduced number of sequences, sites with p-values <0.1 for SLAC, FEL and MEME models, and a posterior probability >0.90 for FUBAR were accepted as candidates for selection [49].

These modules use different methods to estimate ω (dN/ds ratio) at every codon in the alignments and report which codons show evidence of positive or negative selection, using default significance levels. SLAC calculates the expected and observed numbers of synonymous and non-synonymous substitutions to infer selection, whereas FEL directly estimates and applies one ω ratio to all branches. An additional method for detecting pervasive diversifying selection is FUBAR [48], which is similar to FEL. For these analyses, a likelihood ratio test is then used to assess significance. We also tested for the presence of sites with both episodic and pervasive positive selection using MEME [50,51]. This method allows ω to vary across codons as well as across branches of the phylogeny, allowing it to detect a small proportion of branches that are evolving under positive selection [50].

### Estimation of functional divergence of the HIF-α genes

The DIVERGE 3.0 program was used to estimate the Type I and II functional divergence (FD) between HIF-1-3α, and vertebrate/invertebrate orthologs [52,53]. For Type I FD, we used a two-step significance test for rejecting the null hypothesis of no functional divergence (θ = 0), which includes two times the standard error of θ and a likelihood ratio test (critical value = 3.84, df = 1, p < 0.05). For identifying significance with the Type II analysis, pairs with θ values greater than 0 after subtracting two times the standard error were annotated as having undergone functional divergence (p < 0.05, H0: θ = 0) [54].

DIVERGE is only able to estimate divergence at locations where there is no “missing” data in the alignment; therefore, only bHLH and PAS domains were analyzed in 3 of the 4 comparisons, due to the absence of an NTAD or CTAD in certain groups (invertebrates/vertebrates, invertebrates/HIF-1α, invertebrates/HIF-2α), whereas all four domains were analyzed in the other comparison (HIF-1α /HIF-2α).

## Results and discussion

### bHLH+PAS gene family and HIF identification

**Fig 1:**
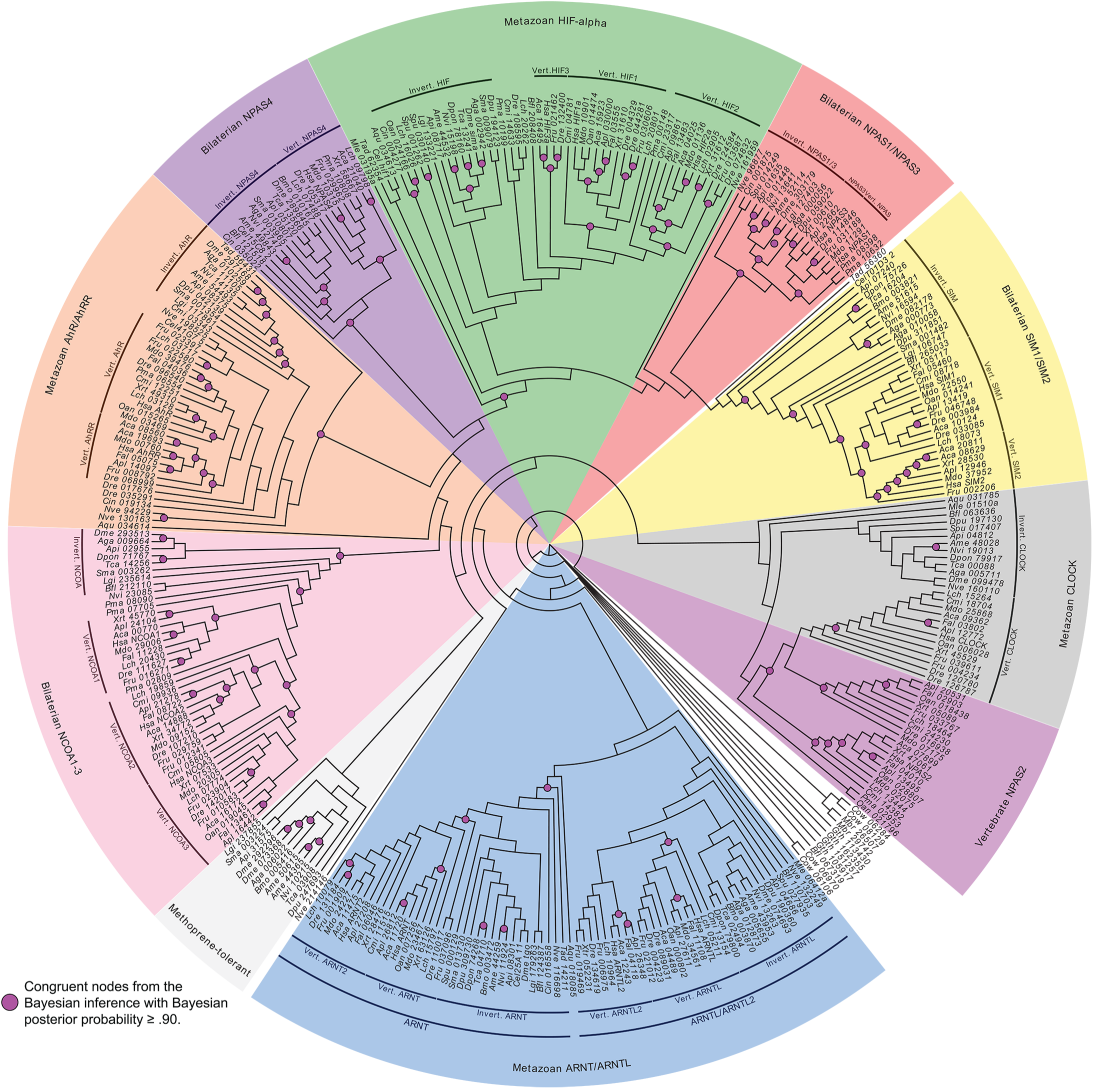
Maximum likelihood tree showing phylogenetic relationships between eukaryotic bHLH+PAS containing proteins. The 10 major clades representing a majority of the bHLH+PAS gene families are highlighted. The names given to each clade are derived from the human gene names found within those clades. For example, bilaterian NPAS1/3 represents a highly supported clade, Bayesian posterior probability (BPP) ≥ 0.90, that contains bilaterian sequences that group with human NPAS1 and human NPAS3. The one exception is the invertebrate-specific clade that contains the *Drosophila melanogaster* methoprene-tolerant gene. The unicellular bHLH+PAS genes typically grouped together. Purple circles indicate congruent nodes between both Bayesian and Maximum likelihood trees with a Bayesian posterior probability support value ≥ 0.90.All support values for this Maximum Likelihood tree, along with Bayesian inference tree and its corresponding support values, are found in S1 and S2 Files.

To evaluate the evolutionary history of the Hypoxia inducible factor (HIF) gene family (both HIF-α and HIF-β genes) and their associated transactivation domains, we searched publicly available eukaryotic genomes for the presence of unique HIF protein domain architecture. HIF genes are part of the larger bHLH+PAS gene family, thus, and thus we initially identified all proteins in each genome that contained a bHLH DNA binding domain plus either one or two PAS domains. To create a phylogeny-based definition of orthology [55,56] we used both Maximum Likelihood and Bayesian inference to generate phylogenetic relationships between the bHLH+PAS domain containing proteins (Fig 1).

In contrast to previous studies, we used Pfam Hidden Markov Models (HMMs) for identification rather than BLAST pairwise similarity searches (BLAST or PSI-BLAST). HMMs are considered more flexible, full probabilistic models for detection of pattern similarities utilizing multiple sequence alignments that can accommodate variable lengths with a focus on domain architecture [57,58].

It is possible that the HMMR model did not recognize specific protein sequences due to significant divergence of the bHLH domain in the genomes searched; however the Pfam bHLH model is based on an alignment of 13,830 sequences using 1,653 species across eukaryotes, suggesting it is a robust domain sequence model. It is also possible that any discordance is due to genome annotation issues, which is a common problem with genomes that have been annotated using computational prediction alone [59–61] resulting in artifacts such as missing or erroneously assigned sequence information [62]. These would present issues for looking at gene family evolution on a micro-evolutionary scale; however, our study design is meant to look at the evolution of this gene family across a wider breadth of animal lineages in an effort to assess macro-evolutionary patterns.

The initial set of bHLH+PAS protein sequences identified clustered into 10 large clades representing major bHLH+PAS gene families including: ARNT and related ARNTL (ARNT/ARNTL), HIF-α 1/2/3, NCOA1-3, AhR/AhRR, NPAS1/3, NPAS2, NPAS4, SIM1/2, CLOCK [21], and an invertebrate-specific gene family that includes the *D*. *melanogaster* gene *methoprene-tolerant* [63,64] with a total of 351 sequences from 35 species (Fig 1). The clade names refer to the human genes found within each clade (except for the *methoprene-tolerant* clade), e.g. human ARNT and human ARNTL are both found within the ARNT/ARNTL clade (Fig 1). Though bHLH domains are present in the genomes of most eukaryotes [65], the specific combination of the bHLH domain with a PAS domain had a more restricted phylogenetic distribution primarily among metazoans with a small number of genes identified in the unicellular bikont *Guillardia theta*, the unicellular filozoan *Capsaspora owczarzaki*, and the choanoflagellate *Monosiga brevicollis* (Fig 1). These unicellular bHLH+PAS genes, however, clustered together and did not group with any metazoan bHLH+PAS genes, except for a single highly divergent *Branchiostoma floridae* gene (Fig 1). These general relationships were inferred through both Bayesian inference and Maximum Likelihood phylogenetic analyses, and we observed mostly congruent topologies between the two methods. From our phylogenetic analyses, we inferred that some bHLH+PAS gene families were absent in non-bilaterian genomes and thus most likely originated in the stem lineage prior to bilaterian diversification. Four bHLH+PAS gene families, AhR/AhRR, CLOCK, ARNT/ARNTL, and HIF-α, were present in at least one of the representative non-bilaterian genomes, suggesting these gene families originated much earlier in metazoan evolution (Fig 1).

**Fig 2:**
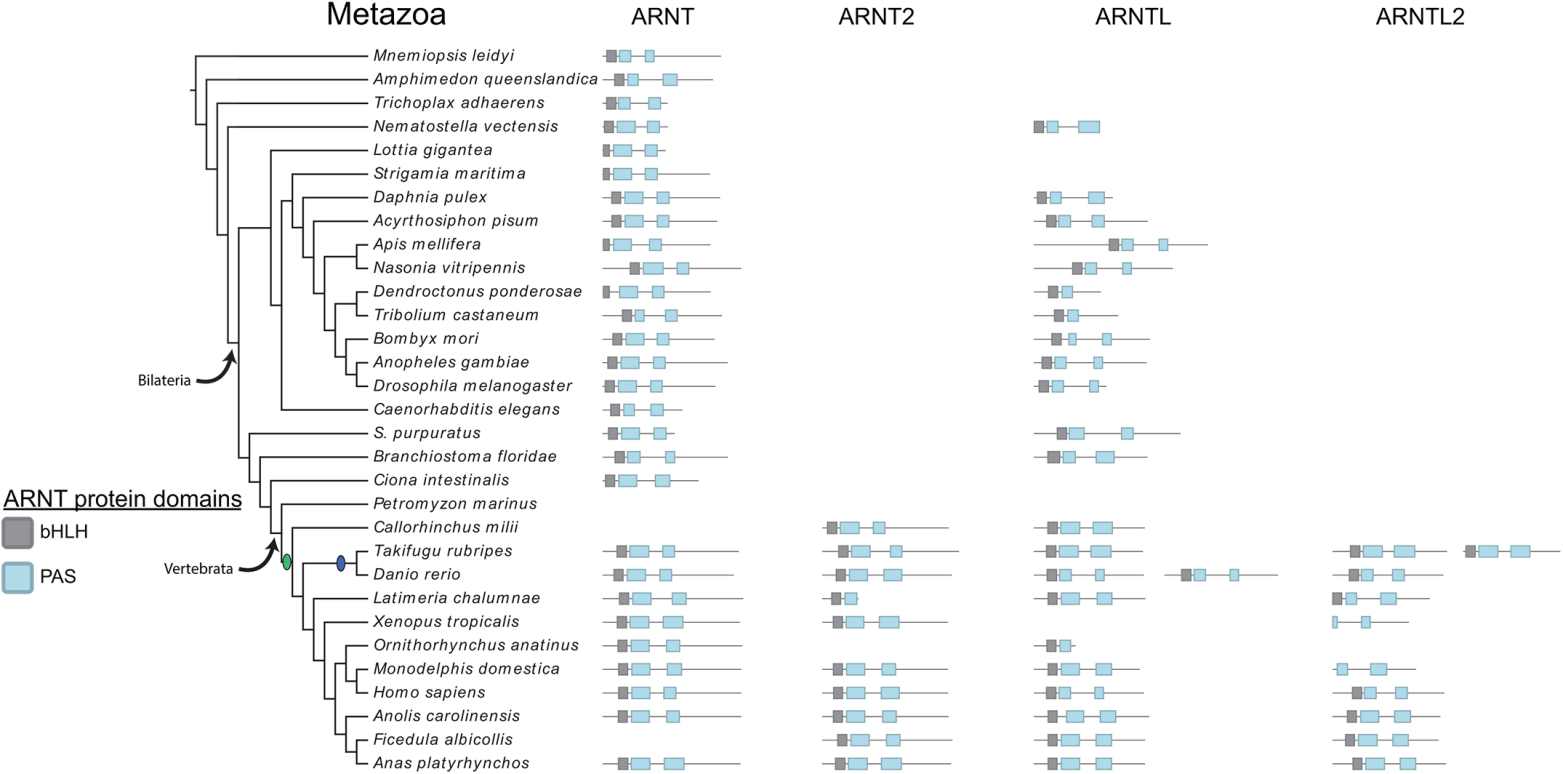
Phylogenetic distribution of the ARNT and ARNTL gene families in Metazoa. Schematics for each ARNT, ARNT2, ARNTL, and ARNTL2 identified in each metazoan species are shown. Black boxes represent the bHLH domains, while the blue boxes represent the PAS domains. Invertebrate genes duplicated to give rise to the different vertebrate paralogs, as a result of the vertebrate genome duplication events (green circle). *Danio rerio* has two ARNTL paralogs, and *Takifugu rubripes* has two ARNTL2 paralogs, both due to the teleost-specific genome duplication event (blue circle). Proteins are drawn to scale. Species phylogenetic relationships are based [35–38].

We recovered an ARNT sequence in all metazoan genomes, except for *Petromyzon marinus* (Figs 1 and 2). Invertebrate ARNTs were phylogenetic distinct from vertebrate ARNTs and an additional vertebrate-specific clade was identified that formed a larger clade with other vertebrate ARNT sequences. This small clade, ARNT2, represents ARNT genes that underwent a round of duplication during the whole genome duplication events in the vertebrate stem lineage (Fig 2). ARNTL proteins have similar protein domain architectures to ARNTs and are phylogenetically related, but are known to functionally interact with different protein families. ARNT subunits mostly dimerize with HIF-α subunits, while ARNTL subunits mostly dimerize with CLOCK proteins. Overall, ARNTL genes duplicated after the vertebrate genome duplication events to form a vertebrate-specific clade of ARNTL2 genes, like the evolutionary pattern seen with the ARNTs (Figs 1 and 2). Interestingly, neither ARNT or ARNT2 duplicates were retained from the teleost-specific duplication event. Additional duplicate paralogs were also seemingly not retained after the two rounds of vertebrate genome duplication events.

**Fig 3:**
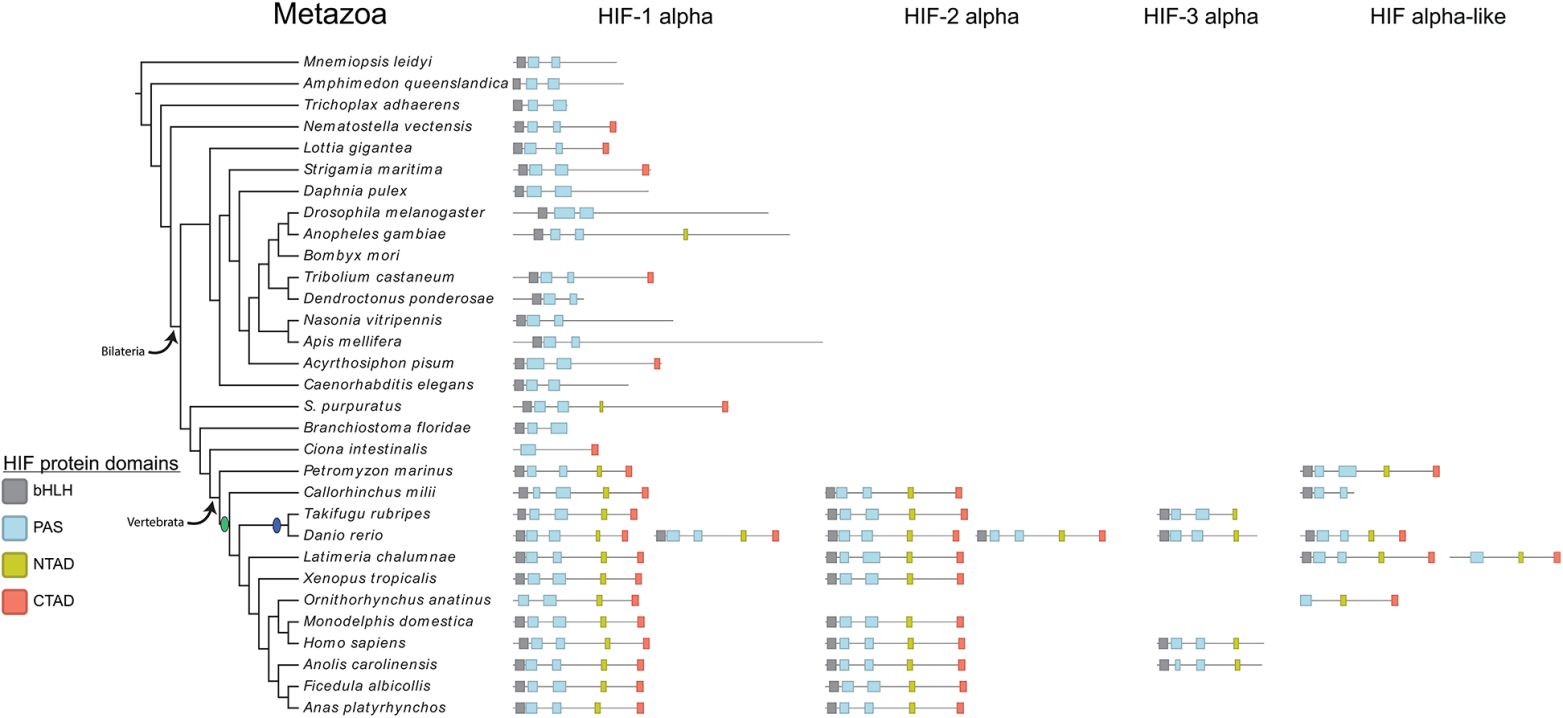
Phylogenetic distribution of the HIF-α genes and associated transactivation domains in Metazoa. Schematics for each HIF-α identified in each metazoan species are shown. Black boxes represent the bHLH domains, blue boxes represent the PAS domains, yellow boxes represent the NTAD, and red boxes represent the CTAD. Invertebrate genes duplicated to give rise to the different vertebrate paralogs, because of the vertebrate genome duplication events (green circle). Additional paralogs of HIF-1α and HIF-2α in *D*. *rerio* are due to the teleost-specific genome duplication event (blue circle). Proteins are drawn to scale. Species phylogenetic relationships are based [35–38].

Similar to the ARNTs, all metazoan genomes, except for *Bombyx mori*, were found to contain at least one HIF-α sequence (Figs 1 and 3). The phylogenetic distribution was again distinct between invertebrate (including non-bilaterians) and vertebrate HIF-α sequences. All non-bilaterian genomes contained one HIF-α, as well as the invertebrate bilaterians. However, up to four HIF-α proteins were identified in vertebrates, with *D*. *rerio* having six (Fig 3). These paralogs resulted from the multiple rounds of genome duplication events in the vertebrate stem lineage. The additional two paralogs seen in *D*. *rerio* were most likely a result from the teleost-specific whole genome duplication. Our phylogenetic analyses recovered three distinct vertebrate HIF-α clades: HIF-1α, HIF-2α, and HIF-3α. Additional vertebrate HIF-α sequences were scattered across the larger HIF-α clade and had a reduced phylogenetic distribution compared to HIF-1α and HIF-2α. These paralogs were classified as HIF-α-like. This suggests that the invertebrate HIF-α duplicated in the vertebrate stem lineage and gave rise to four paralogs, with HIF-1α and HIF-2α being more closely related to each other than to the small HIF-3α clade or the HIF-α-like paralogs. Retention of the teleost specific duplicates was only seen with *D*. *rerio* HIF-1α and HIF-2α. For the most part, it seems that the HIF-3α and HIF-α-like (“HIF-4α”) paralogs were not retained in many vertebrate lineages. Furthermore, it seems that one of each of the teleost-specific HIF-3α and “HIF-4α” paralogs were also not retained. Even so, as seen in Fig 3, the signatures of the 3 rounds of vertebrate genome duplication can be seen in the HIF-α gene family.

### HIF family transactivation domain characteristics

HIF-α proteins, especially vertebrate HIF-1α and HIF-2α, are distinguished by two transactivation domains, the NTAD and CTAD. To understand the separate evolutionary histories associated with these domains, we performed a search for the NTAD and CTAD in the identified HIF-α sequences. In vertebrates, all but one HIF-α sequence contained an NTAD (Fig 3). The CTAD, however, was only found in vertebrate HIF-1α, HIF-2α, and the HIF-α-like sequences (Fig 3). The only exception was the *Callorhinchus milii* HIF-α-like sequence which lacked both an NTAD and CTAD. Within invertebrates, an NTAD was identified in HIF-α sequences of *Strongylocentrotus purpuratus* and *Anopheles gambiae* (Fig 3). The CTAD was found in the HIF-α sequences of the non-bilaterian *N*. *vectensis*, *Lottia gigantea*, *Strigamia maritima*, *Tribolium castaneum*, *Acrythosiphon pisum*, *Strongylocentrotus purpuratus*, *and Ciona intestinalis* (Fig 3).

We failed to identify two characteristic HIF-α domains, the NTAD and CTAD, outside of metazoans. Within metazoans, these two domains were restricted to HIF-α genes. This suggests *de novo* evolution of these domains with HIF-α. The NTAD and CTAD were almost ubiquitous among vertebrate HIF-α genes, but had a more variable distribution among remaining metazoans. Two general scenarios of NTAD and CTAD evolution can be conjectured: (1) the NTAD and CTAD evolved later during bilaterian diversification, and were convergently acquired in a few invertebrates; or (2) the NTAD and CTAD were present in early metazoans, but have been subsequently lost in many invertebrates. Ultimately, the second scenario of NTAD and CTAD loss seems more plausible, because multiple species (*T*. *adhaerens*, *D*. *melanogaster*, *Caenorhabditis elegans*) whose putative HIF orthologs lack both a NTAD and CTAD have been shown to function in hypoxia response [40,66,67]. This functional work along with the widespread absence of the NTAD and CTAD in many HIF-α sequences, suggests that response to hypoxia in many invertebrates might not necessarily require HIF-α function through NTAD- or CTAD-mediated protein-protein interactions, and thus these domains might be dispensable in these invertebrates. In addition, the presence of both the NTAD and CTAD together in most of the vertebrate HIF-1α and HIF-2α sequences could indicate their role in providing a broader protein-protein interaction network enabling a more nuanced regulatory response pathway.

### Selection analyses of the HIF-α genes

Studies in humans have shown that transcription factors and their binding sites evolve quickly [68,69]. Furthermore, transcription factors generally appear to be under greater positive selection as compared to other gene families [70,71]. Analyses of hypoxia-response elements (HREs) observed increased frequencies of HREs in promoter regions of genes in HIF-containing organisms that are under selection [72]. However, our assessment of selective pressures among members of the HIF-α family showed widespread, pervasive purifying selection across their characteristic domains (ω = 0.1684). The number of codons under purifying selection, however, varied slightly across the entire 389 codon-alignment: 274 (SLAC), 309 (FEL), and 320 (FUBAR). None of our analyses revealed statistically significant individual codons under either positive selection (pervasive or episodic) or diversifying selection. Our MEME analysis identified 4 codons with evidence of episodic diversifying selection (codon 158, 223, 313, and 316) (Table 1). In addition, our FEL analysis identified 1 codon with evidence of positive selection (codon 141). These codons are located in the PAS domains (141, 158, 223, and 313) and NTAD (316).

**Table 1:** Summary of results from codon-selection analyses using HYPY.

Selection Analysis	Result
SLAC	274 sites negatively selected ^*a*^
FEL	1 positively selected site ^*a*^^, (codon 141)^
	309 negatively selected sites ^*a*^
FUBAR	0 sites w/ pervasive diversifying selection ^*b*^
	320 sites w/ pervasive purifying selection ^*b*^
MEME	4 sites episodic diversifying selection ^*a*^^, (codons 158, 223, 313, 316)^

*a*– 0.1 significance level

*b*– 0.9 posterior probability

Our site-specific models suggest that the majority of HIF-α domains are under negative selection (i.e. purifying selection), thus they show little variation across the phylogenetic tree. These results were not surprising given the expectation for strong selective pressure to purge variants that would influence DNA binding specificity or protein dimerization (bHLH, PAS domains, respectively); however, the MEME analyses did identify particular sites in a few HIF-α PAS domains under the influence of episodic diversifying selection. Although not entirely conclusive, this result suggests that positive selection may have acted to allow differential accumulation of genetic variation at those sites in different lineages. Overall, our analyses suggest the core of HIF-α sequences/proteins have remained highly conserved over time, with subtle episodic accumulations of advantageous changes in the PAS domain.

We might have expected to recover more evidence of directional or diversifying positive selection in the NTAD and CTAD regions, because they are more likely to cause a functional change via co-factor recruitment and protein-protein interactions. Yet, beyond one test identifying a codon in the NTAD, we were unable to find substantial evidence of positive selection in these domains. It has been suggested that natural selection is predominantly episodic (i.e. containing periods of adaptive evolution), which often is concealed by the prevalence of purifying or neutral selection on other branches [50]. Therefore, it is possible that any initial positive selection regime may have been too transient for our analyses to detect, i.e. most likely an ancient event following the gene duplication in the vertebrate stem lineage.

### Estimation of functional divergence of the HIF-α genes

After a gene duplication event, it is understood that a shift in function, or functional divergence, from ancestral function can occur [73]. We determined estimates for functional divergence (Type I and Type II) among members of the HIF-α gene family. Type I functional divergence usually occurs after gene duplication as a result of relaxed functional constraints between the paralogs via increased genetic variability, resulting in different evolutionary rates between gene clusters. Type II functional divergence is the result of changes in amino acid properties, rather than explicitly altered functional constraints, and are often interpreted as associated with putative functional changes [74,75]. Values of θ that are significantly >0 for either functional divergence test indicates either site-specific altered selective constraints (Type I) or a radical shift in amino acid physiochemical properties after gene duplication (Type II).

Our results support the emergence of the HIF-α gene family functional disparity principally through Type I events. There was widespread occurrence of detectable Type I functional divergence events between invertebrate sequences as compared to vertebrate HIF-α, as well as between vertebrate HIF-1α and HIF-2α. Type I divergence events were substantial across all four domains examined: 73–100% of the bHLH domain, 42–60% of PAS domain, 74% of NTAD and 80% of CTAD domains. This was in stark contrast to the number of codons identified under Type II divergence between invertebrate sequences and vertebrate HIF1-α/HIF-2α sequences with 3.8% of the bHLH domain, 3.5–5% of the PAS domains, 11% of the NTAD domain and 15% of the CTAD domain. Between invertebrate sequences and vertebrate sequences there were no statistically significant codons under Type II divergence.

Thus, our functional divergence results suggest that HIF-α primarily acquired additional structural and/or functional changes, rather than explicit changes in amino acid physiochemical properties, likely due to ancestral constraints. This is demonstrated in both comparisons between (1) invertebrate and vertebrate HIF-α sequences, and (2) vertebrate HIF-1α and HIF-2α, which is in contrast to the lack of evidence of selective pressures (besides purifying selection) across the four characteristic domains. We attribute this to the episodic nature of positive selection, as well as the ability of purifying selection in other lineages to mask these traces. In addition, we were unable to assess such functional divergence in HIF-3α due to its “absence” in most sampled genomes. Following the genome duplication associated with vertebrates, there should be at least 4 HIF-α genes, although it is clear there was a whole-scale loss of some of the paralogs; this is common in gene duplication events, where some duplicates may involve a combination of neofunctionalization and subfunctionalization/gene loss [73]; the latter of which may resemble the fate of HIF-3/”4”α given the obvious absence of a clear phylogenetically related “HIF-4α” group, as well as potentially hinted at by the loss of characteristic domains (CTAD) in HIF-3α.

Overall, this suggests that the relaxed constraint was a major force behind the evolution of HIF-α functional divergence between both invertebrate and vertebrate HIF-α sequences, as well as between vertebrate HIF-1α and HIF-2α sequences, likely due to the challenges associated with oxygen regulation in the different lineages.

## Conclusions

Through the process of inferring the phylogeny of the Hypoxia-Inducible Factor gene family, our results suggest that α-subunits (HIFs) and their β-subunits (ARNTs) evolved at comparable times during metazoan diversification. Putative HIFs and ARNTs were present in most animal genomes of our study, including those of the four non-bilaterians we sampled. The major expansion events for both HIF-α and ARNT gene families were due to the whole genome duplication events in the vertebrate stem lineage, including a teleost specific duplication, with vertebrates having both HIF-1α and HIF-2α paralogs. In contrast, the ARNT family was only represented by two paralogs in vertebrates, most likely due to the duplicated paralogs not being retained over time. ARNTs are hub proteins that are needed by a wide variety of other bHLH+PAS proteins for dimerization. They play a central role for enabling other proteins to exert their regulatory functions, and therefore are potentially under tight evolutionary constraints.

We also assessed the evolution of the HIF family through its characteristic domain repertoire. We show that the NTAD and CTAD domains appear *de novo* within HIFs, with no appearance outside of the HIF-α genes. CTADs first appear in *N*. *vectensis*, have a varied distribution amongst invertebrate bilaterians, and are present in almost every vertebrate HIF-1α and HIF-2α paralog. This suggests a scenario in which the CTAD was acquired *de novo* in the stem lineage after the divergence of *T*. *adhaerens* preceding the diversification of Cnidaria. In this scenario, the CTAD was present in the bilaterian stem lineage, but was subsequently lost in many protostome lineages. Alternatively, the CTAD could have appeared earlier in metazoan diversification, but has since been lost in other extant non-bilaterian animals (e.g. *T*. *adhaerens*). Overall, the lack of genes closely related to HIF-α, or even the lack of bHLH+PAS genes altogether, in almost all unicellular eukaryotes suggests that the innovation of the metazoan HIF gene family could have provided tighter regulation of oxygen homeostasis coinciding with the potential higher oxygen demand in multicellular organisms.

Additionally, we assessed the types of selection potentially at work behind the evolutionary patterns we observed. We find evidence for pervasive purifying selection associated with the bHLH and PAS domains during the expansion and diversification of the HIF-α gene family, with potentially positively selected sites associated with the PAS and NTAD domains; however, overall we found little evidence of positive selection despite strong evidence for Type I functional divergence between vertebrate and invertebrate sequences.

Ultimately, our findings reaffirm that HIF-1α is phylogenetically conserved among most metazoans, whereas HIF-2α appeared later, likely in association with the appearance of specialized systems for O_2_ delivery, such as endothelial vascularization. This is highlighted by our results showing clear functional divergences between HIF-1α and HIF-2α and is accompanied by profound signatures of purifying selection across all four characteristic functional domains. Overall, our findings can be attributed to the substantial integration of this transcription factor family into the critical tasks associated with maintenance of oxygen homeostasis and vascularization, particularly in the vertebrate lineage.

## Supporting information

S1 Table List of sequence IDs for each study species, including the genomic database used for each species. (DOCX)

S1 File Maximum Likelihood tree, generated using PhyML, of the alignment of eukaryotic bHLH+PAS domains. (TRE)

S2 File Bayesian tree, generated using BEAST, of the alignment of concatenated eukaryotic bHLH+PAS domains. (TRE)
