# Supplementary material for: Hypoxia Inducible Factor (HIF) transcription factor family expansion, diversification, divergence and selection in eukaryotes
Source: PLoS One. 2017 Jun 14;12(6):e0179545. doi: 10.1371/journal.pone.0179545 (PMC5470732; doi:10.1371/journal.pone.0179545)
Supplement: S1 Table — (DOCX) [file pone.0179545.s001.docx]

| ***Species Name*** | **Prefix** | **bHLH+PAS sequences** | **Database** | **Sequence IDs** |
| --- | --- | --- | --- | --- |
| *Anas platyrhynchos* | Apl | Apl_09861  Apl_12772  Apl_12946  Apl_13419  Apl_13495  Apl_14097  Apl_16442  Apl_20531  Apl_21278  Apl_22662  Apl_24104  Apl_26348  Apl_26812  Apl_27208  Apl_27611  Apl_30000 | NCBI | XP_005009861  XP_005012772  XP_005012946  XP_005013419  XP_005013495  XP_005014097  XP_005016442  XP_005020531  XP_005021278  XP_005022662  XP_005024104  XP_005026348  XP_005026812  XP_005027208  XP_005027611  XP_005030000 |
| *Ficedula albicollis* | Fal | Fal_02903  Fal_03555  Fal_03802  Fal_04010  Fal_04036  Fal_04118  Fal_05075  Fal_05460  Fal_06046  Fal_08722  Fal_11108  Fal_11228  Fal_13461  Fal_13483 | Ensembl | ENSFALP00000002903  ENSFALP00000003555  ENSFALP00000003802  ENSFALP00000004010  ENSFALP00000004036  ENSFALP00000004118  ENSFALP00000005075  ENSFALP00000005460  ENSFALP00000006046  ENSFALP00000008722  ENSFALP00000011108  ENSFALP00000011228  ENSFALP00000013461  ENSFALP00000013483 |
| *Anolis carolinensis* | Aca | Aca_00770  Aca_04025  Aca_04489  Aca_07899  Aca_08560  Aca_08629  Aca_09362  Aca_10124  Aca_11475  Aca_11720  Aca_12243  Aca_14888  Aca_15923  Aca_16172  Aca_16495  Aca_19693  Aca_20811  Aca_21040 | Ensembl | ENSACAP00000000770  ENSACAP00000004025  ENSACAP00000004489  ENSACAP00000007899  ENSACAP00000008560  ENSACAP00000008629  ENSACAP00000009362  ENSACAP00000010124  ENSACAP00000011475  ENSACAP00000011720  ENSACAP00000012243  ENSACAP00000014888  ENSACAP00000015923  ENSACAP00000016172  ENSACAP00000016495  ENSACAP00000019693  ENSACAP00000020811  ENSACAP00000021040 |
| *Monodelphis domestica* | Mdo | Mdo_00760  Mdo_01136  Mdo_02075  Mdo_03469  Mdo_07175  Mdo_09152  Mdo_10315  Mdo_10901  Mdo_11291  Mdo_20305  Mdo_22550  Mdo_23467  Mdo_24227  Mdo_25868  Mdo_29006  Mdo_34561  Mdo_37952  Mdo_39476 | Ensembl | ENSMODP00000000760  ENSMODP00000001136  ENSMODP00000002075  ENSMODP00000003469  ENSMODP00000007175  ENSMODP00000009152  ENSMODP00000010315  ENSMODP00000010901  ENSMODP00000011291  ENSMODP00000020305  ENSMODP00000022550  ENSMODP00000023467  ENSMODP00000024227  ENSMODP00000025868  ENSMODP00000029006  ENSMODP00000034561  ENSMODP00000037952  ENSMODP00000039476 |
| *Ornithorhynchus anatinus* | Oan | Oan_000802  Oan_006028  Oan_007256  Oan_014241  Oan_014474  Oan_015265  Oan_018438  Oan_019045  Oan_021796  Oan_024166  Oan_028807 | Ensembl | ENSOANP00000000802  ENSOANP00000006028  ENSOANP00000007256  ENSOANP00000014241  ENSOANP00000014474  ENSOANP00000015265  ENSOANP00000018438  ENSOANP00000019045  ENSOANP00000021796  ENSOANP00000024166  ENSOANP00000028807 |
| *Xenopus tropicalis* | Xtr | Xrt_00610  Xrt_05089 Xrt_05117 Xrt_07533 Xrt_28476 Xrt_28530 Xrt_31610 Xrt_31612 Xrt_34772 Xrt_45529 Xrt_45770 Xrt_47061 Xrt_49310 Xrt_58667 Xrt_63126 | Ensembl | ENSXETP00000000610  ENSXETP00000005089  ENSXETP00000005117  ENSXETP00000007533  ENSXETP00000028476  ENSXETP00000028530  ENSXETP00000031610  ENSXETP00000031612  ENSXETP00000034772  ENSXETP00000045529  ENSXETP00000045770  ENSXETP00000047061  ENSXETP00000049310  ENSXETP00000058667  ENSXETP00000063126 |
| *Latimeria chalumnae* | Lch | Lch_02905  Lch_03128  Lch_03717  Lch_05211  Lch_07488  Lch_07774  Lch_09798  Lch_10079  Lch_10964  Lch_13444  Lch_15264  Lch_15797  Lch_16026  Lch_18073  Lch_18464  Lch_19859  Lch_20262  Lch_20430  Lch_20801 | Ensembl | ENSLACP00000002905  ENSLACP00000003128  ENSLACP00000003717  ENSLACP00000005211  ENSLACP00000007488  ENSLACP00000007774  ENSLACP00000009798  ENSLACP00000010079  ENSLACP00000010964  ENSLACP00000013444  ENSLACP00000015264  ENSLACP00000015797  ENSLACP00000016026  ENSLACP00000018073  ENSLACP00000018464  ENSLACP00000019859  ENSLACP00000020262  ENSLACP00000020430  ENSLACP00000020801 |
| *Danio rerio* | Dre | Dre_003984  Dre_004223  Dre_004329  Dre_016938  Dre_017676  Dre_033085  Dre_035291  Dre_044281  Dre_068999  Dre_074832  Dre_089031  Dre_096540  Dre_105317  Dre_107210  Dre_108595  Dre_110017  Dre_111627  Dre_114846  Dre_120780  Dre_124584  Dre_126787  Dre_131184  Dre_132400  Dre_134619  Dre_142017 | Ensembl | ENSDARP00000003984  ENSDARP00000004223  ENSDARP00000004329  ENSDARP00000016938  ENSDARP00000017676  ENSDARP00000033085  ENSDARP00000035291  ENSDARP00000044281  ENSDARP00000068999  ENSDARP00000074832  ENSDARP00000089031  ENSDARP00000096540  ENSDARP00000105317  ENSDARP00000107210  ENSDARP00000108595  ENSDARP00000110017  ENSDARP00000111627  ENSDARP00000114846  ENSDARP00000120780  ENSDARP00000124584  ENSDARP00000126787  ENSDARP00000131184  ENSDARP00000132400  ENSDARP00000134619  ENSDARP00000142017 |
| *Takifugu rubripes* | Fru | Fru_001909  Fru_002206  Fru_004234  Fru_006975  Fru_008792  Fru_010583  Fru_012341  Fru_013587  Fru_016271  Fru_019469  Fru_021012  Fru_022580  Fru_023397  Fru_023904  Fru_029759  Fru_029962  Fru_030606  Fru_031189  Fru_033767  Fru_037096  Fru_039611  Fru_046748_ |  | ENSTRUP00000001909  ENSTRUP00000002206  ENSTRUP00000004234  ENSTRUP00000006975  ENSTRUP00000008792  ENSTRUP00000010583  ENSTRUP00000012341  ENSTRUP00000013587  ENSTRUP00000016271  ENSTRUP00000019469  ENSTRUP00000021012  ENSTRUP00000022580  ENSTRUP00000023397  ENSTRUP00000023904  ENSTRUP00000029759  ENSTRUP00000029962  ENSTRUP00000030606  ENSTRUP00000031189  ENSTRUP00000033767  ENSTRUP00000037096  ENSTRUP00000039611  ENSTRUP00000046748 |
| *Callorhinchus milli* | Cmi | Cmi_03343  Cmi_04781  Cmi_05805  Cmi_08718  Cmi_09936  Cmi_11515  Cmi_12251  Cmi_13154  Cmi_14382  Cmi_14633  Cmi_18704  Cmi_23317  Cmi_24230 | Ensembl | SINCAMP00000003343  SINCAMP00000004781  SINCAMP00000005805  SINCAMP00000008718  SINCAMP00000009936  SINCAMP00000011515  SINCAMP00000012251  SINCAMP00000013154  SINCAMP00000014382  SINCAMP00000014633  SINCAMP00000018704  SINCAMP00000023317  SINCAMP00000024230 |
| *Petromyzon marinus* | Pma | Pma_00148  Pma_02809  Pma_02953  Pma_06554  Pma_07705  Pma_08090  Pma_08398  Pma_10199  Pma_10632  Pma_10808 | Ensembl | ENSPMAP00000000148  ENSPMAP00000002809  ENSPMAP00000002953  ENSPMAP00000006554  ENSPMAP00000007705  ENSPMAP00000008090  ENSPMAP00000008398  ENSPMAP00000010199  ENSPMAP00000010632  ENSPMAP00000010808 |
| *Ciona intestinalis* | Cin | Cin_001875  Cin_004277  Cin_016558  Cin_019134  Cin_035022 | Ensembl | ENSCINP00000001875  ENSCINP00000004277  ENSCINP00000016558  ENSCINP00000019134  ENSCINP00000035022 |
| *Branchiostoma floridae* | Bfl | Bfl_063636  Bfl_067319  Bfl_110703  Bfl_121518  Bfl_124387  Bfl_208408  Bfl_212110  Bfl_265033 | JGI | jgi\|Brafl1\|63636\|fgenesh2_pg.scaffold_2000065  jgi\|Brafl1\|67319\|fgenesh2_pg.scaffold_14000012  jgi\|Brafl1\|110703\|fgenesh2_pg.scaffold_792000016  jgi\|Brafl1\|121518\|estExt_fgenesh2_pg.C_680040  jgi\|Brafl1\|124387\|estExt_fgenesh2_pg.C_146006  jgi\|Brafl1\|208408\|e_gw.35.42.1  jgi\|Brafl1\|212110\|e_gw.47.74.1  jgi\|Brafl1\|265033\|estExt_GenewiseH_1.C_790104 |
| *Strongylocentrotus purpuratus* | Spu | Spu_000129  Spu_001262  Spu_014249  Spu_017407  Spu_027935  Spu_030140 | Ensembl | SPU_000129-tr  SPU_001262-tr  SPU_014249-tr  SPU_017407-tr  SPU_027935-tr  SPU_030140-tr |
| *Lottia gigantea* | Lgi | Lgi_106747  Lgi_117861  Lgi_127403  Lgi_133221  Lgi_179263  Lgi_235614  Lgi_237855 | JGI | jgi\|Lotgi1\|106747\|e_gw1.5.208.1  jgi\|Lotgi1\|117861\|e_gw1.27.139.1  jgi\|Lotgi1\|127403\|e_gw1.57.263.1  jgi\|Lotgi1\|133221\|e_gw1.83.92.1  jgi\|Lotgi1\|179263\|fgenesh2_pm.C_sca_58000008  jgi\|Lotgi1\|235614\|estExt_fgenesh2_pg.C_sca_670068  jgi\|Lotgi1\|237855\|estExt_fgenesh2_pg.C_sca_1200037 |
| *Caenorhabditis elegans* | Cel | Cel15C8_2  Cel25A_11  Cel41G7_5  Cel_hif1  CelT01D3_2 | Ensembl | C15C8.2  C25A1.11  C41G7.5  F38A6.3  T01D3.2 |
| *Strigamia maritima* | Sma | Sma_001354  Sma_001482  Sma_003224  Sma_003262  Sma_009079  Sma_010807  Sma_013730 | Ensembl | SMAR001354-PA  SMAR001482-PA  SMAR003224-PA  SMAR003262-PA  SMAR009079-PA  SMAR010807-PA  SMAR013730-PA |
| *Daphnia pulex* | Dpu | Dpu_042139  Dpu_059022  Dpu_190360  Dpu_194123  Dpu_197130  Dpu_247693  Dpu_309730  Dpu_311851 | JGI | DappuP042139  DappuP059022  DappuP190360  DappuP194123  DappuP197130  DappuP247693  DappuP309730  DappuP311851 |
| *Acyrthosiphon pisum* | Api | Api_01635  Api_02955  Api_04686  Api_04812  Api_05120  Api_07240  Api_08301  Api_08375  Api_08717  Api_31575 | Ensembl | ACYPI001635  ACYPI002955  ACYPI004686  ACYPI004812  ACYPI005120  ACYPI007240  ACYPI008301  ACYPI008375  ACYPI008717  ACYPI031575 |
| *Apis mellifera* | Ame | Ame_43953  Ame_44259  Ame_44365  Ame_44532  Ame_48028  Ame_49843  Ame_50615  Ame_51615  Ame_52114  Ame_54493 | Ensembl | GB43953  GB44259  GB44365  GB44532  GB48028  GB49843  GB50615  GB51615  GB52114  GB54493 |
| *Nasonia vitripennis* | Nvi | Nvi_10215  Nvi_11924  Nvi_12747  Nvi_13263  Nvi_13641  Nvi_14135  Nvi_15198  Nvi_16594  Nvi_19013  Nvi_23085 | Ensembl | NV10215  NV11924  NV12747  NV13263  NV13641  NV14135  NV15198  NV16594  NV19013  NV23085 |
| *Dendroctonus ponderosae* | Dpon | Dpon_71767  Dpon_74268  Dpon_75726  Dpon_78160  Dpon_79800  Dpon_79917 | Ensembl | ENN71767  ENN74268  ENN75726  ENN78160  ENN79800  ENN79917 |
| *Tribolium castaneum* | Tca | Tca_00088  Tca_01448  Tca_02494  Tca_03908  Tca_04710  Tca_11105  Tca_13241  Tca_13566  Tca_14256  Tca_16204 | Ensembl | TC000088  TC001448  TC002494  TC003908  TC004710  TC011105  TC013241  TC013566  TC014256  TC016204 |
| *Bombyx mori* | Bmo | Bmo_003472  Bmo_003821  Bmo_003870  Bmo_005416  Bmo_010167 | Ensembl | BGIBMGA003472  BGIBMGA003821  BGIBMGA003870  BGIBMGA005416  BGIBMGA010167 |
| *Anopheles gambiae* | Aga | Aga_000056  Aga_000773  Aga_002942  Aga_003965  Aga_005655  Aga_005711  Aga_006022  Aga_009664  Aga_010058  Aga_010259  Aga_012873 | Ensembl | AGAP000056  AGAP000773  AGAP002942  AGAP003965  AGAP005655  AGAP005711  AGAP006022  AGAP009664  AGAP010058  AGAP010259  AGAP012873 |
| *Drosophila melanogaster* | Dme | Dme_073368  Dme_074693  Dme_082178  Dme_099478  Dme_289845  Dme_292296  Dme_293513  Dme_297168  Dme_303179  Dme_sima  Dme_tgo | Flybase | FBpp0073368  FBpp0074693  FBpp0082178  FBpp0099478  FBpp0289845  FBpp0292296  FBpp0293513  FBpp0297168  FBpp0303179  FBpp0310749  FBpp0081483 |
| *Nematostella vectensis* | Nve | Nve_94229  Nve_96877  Nve_116698  Nve_130163  Nve_132249  Nve_160110  Nve_161959  Nve_198525  Nve_214146 | JGI | jgi\|Nemve1\|94229\|e_gw.35.237.1  jgi\|Nemve1\|96877\|e_gw.44.108.1  jgi\|Nemve1\|116698\|e_gw.140.42.1  jgi\|Nemve1\|130163\|e_gw.274.25.1  jgi\|Nemve1\|132249\|e_gw.307.53.1  jgi\|Nemve1\|160110\|estExt_gwp.C_120110  jgi\|Nemve1\|161959\|estExt_gwp.C_260085  jgi\|Nemve1\|198525\|fgenesh1_pg.scaffold_10000117  jgi\|Nemve1\|214146\|fgenesh1_pg.scaffold_193000011 |
| *Trichoplax adhaerens* | Tad | Tad_14211  Tad_56360  Tad_56431  Tad_62354 | JGI | jgi\|Triad1\|14211\|gw1.3.1240.1  jgi\|Triad1\|56360\|fgeneshTA2_pg.C_scaffold_5000254  jgi\|Triad1\|56431\|fgeneshTA2_pg.C_scaffold_5000325  jgi\|Triad1\|62354\|fgeneshTA2_pg.C_scaffold_42000010 |
| *Amphimedon queenslandica* | Aqu | Aqu_018085  Aqu_031785  Aqu_034613  Aqu_034614 | NCBI | XP_011406878.1  XP_003386656.1  XP_011403285.1  XP_011403286.1 |
| *Mnemiopsis leidyi* | Mle | Mle_01510a  Mle_03195a  Mle_06412a | Mnemiopsis Genome Portal | ML01510a  ML03195a  ML06412a |
| *Monosiga brevicollis* | Mbr | Mbr_26507  Mbr_29742 | JGI | jgi\|Monbr1\|26507\|fgenesh2_pg.scaffold_14000173  jgi\|Monbr1\|29742\|fgenesh2_pg.scaffold_38000023 |
| *Capsaspora owczarzaki* | Cow | Cow_06106  Cow_06284  Cow_06970  Cow_08129 | Ensembl | CAOG_006106  CAOG_006284  CAOG_006970  CAOG_008129 |
| *Guillardia theta* | Gth | Gth_105917  Gth_113430  Gth_151257  Gth_162355 | JGI | jgi\|Guith1\|105917\|au.22_g7019  jgi\|Guith1\|113430\|au.67_g14532  jgi\|Guith1\|151257\|fgenesh2_kg.15_#_37_#_1730_1_CCFI_CCFN_EXTA_EXTB  jgi\|Guith1\|162355\|estExt_fgenesh2_pg.C_200195 |
